# Cutaneous-Tropism Viruses: Unraveling Pathogenetic Mechanisms and Immunoprophylactic Strategies

**DOI:** 10.3390/life16010174

**Published:** 2026-01-21

**Authors:** Mariana Lupoae, Alina Mihaela Elisei, Ancuța Iacob, Andreea Lupoae, Alin Laurențiu Tatu, Elena Niculeț, Maria Nina Căuș, Denisa Batîr, Aurel Nechita, Mădălina Nicoleta Matei, Claudia Simona Ștefan, Elena Lăcrămioara Lisă, Lungu Irinel, Dana Tutunaru

**Affiliations:** 1 Research Centre in the Medical-Pharmaceutical Field, Department of Pharmaceutical Science, Faculty of Medicine and Pharmacy, “Dunarea de Jos” University of Galati, 800008 Galati, Romania; mariana.lupoae@ugal.ro (M.L.); aelisei@ugal.ro (A.M.E.); maria.caus@ugal.ro (M.N.C.); denisa.batir@ugal.ro (D.B.); claudia.stefan@ugal.ro (C.S.Ș.); elena.lisa@ugal.ro (E.L.L.); irinel.lungu@ugal.ro (L.I.); dana.tutunaru@ugal.ro (D.T.); 2 “Sf. Apostol Andrei” County Emergency Clinical Hospital, 800578 Galati, Romania; 3 Clinical Medical Department, Faculty of Medicine and Pharmacy, “Dunarea de Jos” University of Galati, 800008 Galati, Romania; alin.tatu@ugal.ro (A.L.T.); nechitaaurel@yahoo.com (A.N.); 4 Department of Morphological and Functional Sciences, Faculty of Medicine and Pharmacy, “Dunarea de Jos” University of Galati, 800008 Galati, Romania; elena.niculet@ugal.ro; 5 Dental Medicine Departament, “Dunarea de Jos” University of Galati, 800008 Galati, Romania; madalina.matei@ugal.ro

**Keywords:** viral infections, cutaneous manifestations, viral tropism, treatment, prophylaxis

## Abstract

Cutaneous viral infections result from the complex interaction between viruses and skin structures, influenced by viral tropism and the host immune response. They can generate lesions ranging from transient rashes to chronic or potentially tumorous formations. Cutaneous manifestations are often the first sign of infection and allow for early recognition. The aim of this review is to analyze the role of viruses in skin pathology, the mechanisms of infection, and the clinical impact. A narrative review of the recent literature was performed, including original articles, systematic reviews, and clinical guidelines on cutaneous viral infections. Data on pathogenic mechanisms, types of lesions, evolution, and therapeutic options were evaluated, covering the main viruses involved in dermatology: herpesviruses, papillomaviruses, poxviruses, and viruses associated with acute rashes. Cutaneous viral infections can be self-limited, recurrent, or chronic, and some can promote malignant transformation of skin cells. The variability of clinical manifestations reflects the virus–host interaction and influences diagnosis and management. Recent advances highlight the development of vaccines and targeted antiviral therapies, which improve prognosis and infection control. Viruses play a major role in the etiology of skin diseases, and their early recognition is essential for preventing complications. Understanding the mechanisms of infection and the cutaneous response contributes to the optimization of therapeutic and preventive strategies, strengthening the modern management of viral cutaneous pathology.

## 1. Introduction

**Figure 1:**
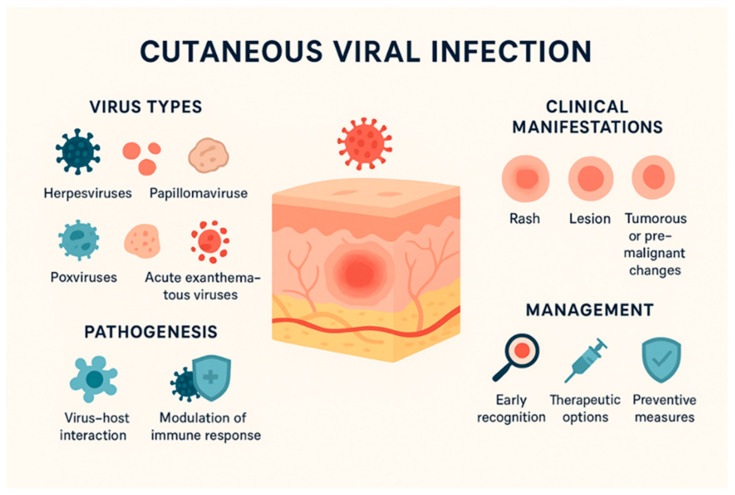
A cross-section of skin with a viral lesion, highlighting how viruses affect the skin layer (Image generated using the ChatGPT-4 model on 12 September 2025).

Over the past two decades, the world has been under siege by various viral epidemics and pandemics, which have disrupted the health of millions of people and caused countless deaths [1]. Viruses are subcellular, infectious biological entities, visible only under an electron microscope, that obligately parasitize living cells. They belong to a separate kingdom, Vira, with over 4000 viruses identified to date. These biological agents can infect prokaryotic or eukaryotic cells, including those of fungi, plants, and animals [2,3]. The first viral pathogens were isolated from plants, such as tobacco mosaic virus, discovered by Ivanovsky in 1892. Later, viruses were also identified in animals and humans. The discovery of avian sarcoma virus (Rous virus) by Peyton Rous in 1911 first highlighted the oncogenic potential of some viruses [4,5]. Viruses are acellular structures, very small in size (18–300 nm), filterable and ultrafilterable. Their genome contains a single type of nucleic acid, deoxyribonucleic acid (DNA) or ribonucleic acid (RNA). They are obligate intracellular parasites and can only multiply in living cells, and cannot be cultivated on ordinary nutrient media. Viruses lack ribosomes, are not sensitive to antibiotics, and have biological specificity, with each virus usually causing a specific disease [6]. Vaccines induce active immunity, which gives the body a specific resistance to diseases. Specific antiviral prophylaxis can be achieved in two ways: active and passive. The protection obtained after vaccination is due to both humoral immunity, through the production of antibodies, and cellular immunity, through the formation of sensitized lymphocytes, both generated in response to the administration of vaccine antigens [7,8]. Passive prophylaxis consists of the direct administration of antibodies, generating a form of humoral protection. Immunoglobulins maintain their effectiveness when administered both before exposure and after the onset of infection, contributing to the prevention of the development of the disease [9]. This review aims to systematize the main viruses involved in skin pathology and the mechanisms by which they generate dermatological manifestations, as well as to evaluate current prevention options, with an emphasis on immunoprophylaxis and modern vaccination strategies. Understanding the general mechanisms of viral skin infection is essential for elucidating pathogenesis, developing therapeutic interventions and prevention strategies, and identifying knowledge gaps and future directions for research in dermatovirology (Figure 1).

## 2. Materials

**Figure 2:**
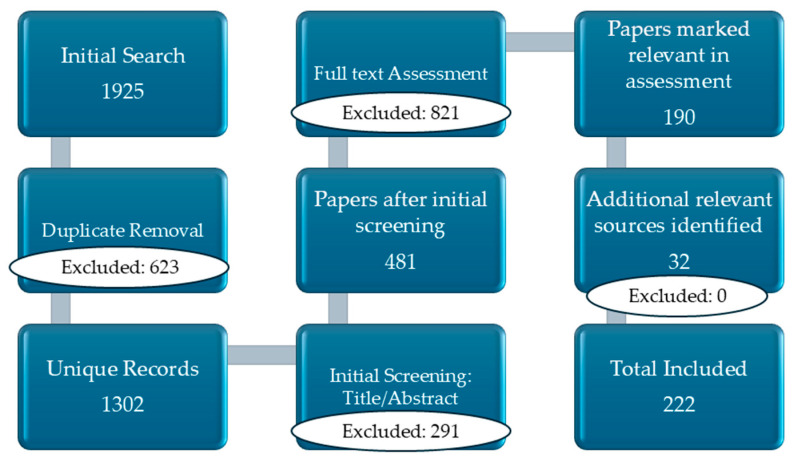
PRISMA flow diagram of the study selection process.

This narrative review was conducted to synthesize current evidence from major biomedical databases (PubMed, Scopus, Web of Science) for publications in English up to December 2025. The search string utilized combinations of Boolean operators and keywords: “viral infections” OR “viral tropism” AND “cutaneous manifestations” AND “treatment” OR “prophylaxis”. Additional references were identified from the bibliographies of relevant articles and official reports from international health agencies, such as the World Health Organization (WHO) and the European Centre for Disease Prevention and Control (ECDC). Article selection was based on relevance, originality, and contribution to the analysis and synthesis of current knowledge regarding the involvement of viruses in cutaneous pathology, highlighting mechanisms of infection, associated lesion types, clinical impact, and therapeutic perspectives. Both original research and review articles, as well as recent epidemiological reports and policy documents, were considered. Particular attention was given to studies addressing the importance of virus-specific recognition in the diagnosis and management of dermatological conditions. The evidence was synthesized to provide an integrated and up-to-date overview, aiming to highlight knowledge gaps and future research directions. Figure 2 illustrates the PRISMA flow diagram, detailing the systematic process of study identification and the application of inclusion and exclusion criteria. Inclusion criteria were (1) original research (clinical trials, cohort studies, case–control studies); (2) comprehensive review articles and meta-analyses; (3) recent epidemiological reports and policy documents; and (4) studies focusing on pathophysiological mechanisms or therapeutic perspectives. Exclusion criteria were (1) articles not available in English; (2) studies with insufficient clinical data; and (3) redundant reports or those not peer-reviewed (except for official agency reports). A total of 1925 records were initially identified. Following the removal of duplicates, 1302 unique entries underwent title and abstract screening, with 481 proceeding to full-text eligibility review. After excluding 291 reports that did not meet the inclusion criteria, 190 studies remained. By screening reference lists and consulting experts, 32 further sources were added, resulting in a final sample of 222 studies. During the preparation of this manuscript, ChatGPT, version 4, was used for linguistic editing, text structuring, and figure preparation.

## 3. Skin Barriers and Viral Penetration Mechanisms

The skin is the first line of defense against pathogens, including viruses, through the epidermal barrier, corneocyte cohesion, and the local immune system [10,11]. Structures such as the stratum corneum, epidermal lipids, and intercellular junctions effectively limit viral access [12]. The stratum corneum, composed of corneocytes bonded to a lipid matrix, limits viral entry through its structural integrity and acidic pH (acid mantle), which inhibits the stability of many viral particles. Tight junctions (claudin-1, occludin, ZO-1) located in the granular layer limit paracellular penetration and protect access to viral receptors in the deeper layers [13]. Langerhans cells, keratinocytes, and antimicrobial peptides contribute to the immunological barrier, limiting viral attachment and initial replication [14]. Antimicrobial peptides (defensins, cathelicidins), the skin microbiome, and keratinocyte turnover also constitute effective protective mechanisms. To initiate infection, viruses must overcome these barriers. Penetration usually occurs at the level of continuity solutions (microtraumas, abrasions, cracks), through hair follicles, sebaceous glands, or mucous membranes adjacent to skin areas [15,16]. Viruses cannot generally infect intact skin; skin infection occurs predominantly in conditions of barrier dysfunction (dermatitis, inflammation, microlesions) [17].

In atopic dermatitis, alterations in filaggrin and reduced expression of tight junctions facilitate herpes simplex virus 1 (HSV-1) entry. Some agents, such as papillomaviruses, require exposure of basal cells of the epidermis by local trauma, whereas herpes simplex viruses use specific receptors on keratinocytes even in the case of minimal damage [18]. The viruses enter keratinocytes or other skin cells, multiply, and cause cell destruction, leading to the appearance of vesicles, papules, or ulcers. Langerhans cells, keratinocytes, and antimicrobial peptides contribute to the immunological barrier, limiting viral attachment and initial replication [12]. HSV-1 uses the Nectin-1 receptor on keratinocytes; in normal skin this receptor is masked by functional junctions, becoming accessible only under conditions of epidermal disruption. Viral entry occurs through rapid membrane fusion, a Nectin-1-dependent process [19]. Immune-induced inflammation can aggravate skin lesions by recruiting lymphocytes and macrophages, producing erythema and edema. Superficial microcracks or abrasions can expose cellular receptors, although the stratum corneum and junctions still remain partially protective [20]. Some viruses (e.g., herpesviruses) remain latent in ganglia or skin cells, causing recurrences of lesions. Certain viruses (e.g., human papillomavirus (HPV)) can alter the cell cycle and growth control mechanisms, favoring uncontrolled proliferation and, over time, the appearance of skin tumors [21]. The cytokines IL-4 and IL-13, characteristic of Th2-type inflammation, reduce epidermal cohesion and facilitate viral access. Intact skin prevents viral skin infections, including HSV-1 and Varicella-Zoster Virus (VZV). Barrier compromise favors disseminated infections, especially in atopic dermatitis or immunosuppression. Understanding these mechanisms guides therapeutic interventions aimed at restoring the barrier and preventing viral skin infections [16].

### 3.1. Viral Tropism for Epidermal and Dermal Cells

Viral tropism refers to the range of cells and tissues in which a virus can establish a productive infection. For a cell to be susceptible, it must express virus-specific entry receptors and be permissive for viral replication, and the virus must be able to evade the local immune response [22]. In human skin, the main target cells are epidermal keratinocytes, dermal endothelial cells, and nerve endings, each of which causes specific clinical manifestations [23].

Cellular receptor expression is dynamic and can be influenced by inflammation, trauma, coinfection, or changes in the skin microbiome. The proliferative and metabolic status of keratinocytes influences viral replication, and the local immune system can prevent or facilitate infection [24].

Epithelial tropism explains verrucous and papular lesions, neurotropic tropism explains viral latency and recurrences, and vascular tropism explains exanthematous and purpuric manifestations [25]. Understanding viral tropism is essential for the development of antiviral therapies, vaccines, and experimental studies.

Tropism is determined by cellular expression, namely the compatibility of viral replication mechanisms with cellular metabolism, and the viral capacity of viral receptors to bypass local defense mechanisms [26]. Epitheliotropic viruses infect epidermal keratinocytes. Examples include HPV, molluscum contagiosum virus (MCV), and herpes simplex virus (HSV) [27]. Neurotropic viruses initially infect epithelial cells, then migrate to sensory ganglia, establishing latency and causing subsequent cutaneous reactivations, as in the case of HSV and VZV [28,29]. Melanocytes, although not the primary target of VZV infection, may be secondarily affected by epidermal infection and local inflammation, contributing to dysfunction of the melano-keratinocyte unit and explaining post-zoster pigmentation disorders [30].

Vasculotropic viruses infect dermal endothelial cells, causing maculopapular or purpuric lesions, e.g., Parvovirus B19 [31].

**Figure 3:**
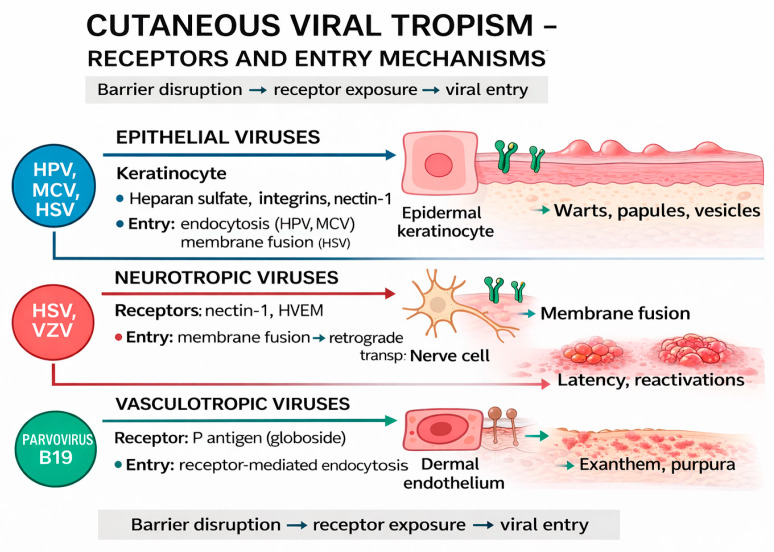
Schematic representation of cutaneous viral tropism, highlighting the binding of viruses to cell surface receptors and the main entry mechanisms, correlated with the type of cells infected and the resulting clinical manifestations. (Image generated using the ChatGPT-4 model on 12 September 2025).

Figure 3 illustrates, in a simplified form, how different skin-tropic viruses affect the skin depending on the type of cells infected. Viruses initially interact by binding to specific cell surface receptors, followed by distinct entry mechanisms, such as receptor-mediated endocytosis, membrane fusion, or neuronal transport. The integration of surface receptors and entry pathways explains the relationship between cell tropism and characteristic skin clinical manifestations.

Thus, epithelial viruses (HPV, MCV, HSV) predominantly infect epidermal keratinocytes, leading to the appearance of warts and papules, as a result of local cell proliferation [32,33]. Neurotropic viruses (HSV, VZV) have an affinity for nerve cells, where they can establish latent infections, with the possibility of periodic reactivation, clinically manifested by recurrent vesicular lesions distributed dermatomally [34]. In contrast, vasculotropic viruses, represented in particular by Parvovirus B19, affect the dermal endothelium, causing exanthemas and purpura, as an expression of vascular involvement and systemic immune response [35].

Therefore, the distribution of receptors and the metabolic status of cells influence the susceptibility of skin tissue. Also, the presence of inflammation, concomitant infections, or skin dysbiosis can modify viral tropism. Viral tropism for epidermal and dermal cells results from the complex interaction between virus and host, influenced by cellular receptors, metabolic status, immune evasion, and the skin microenvironment. This determines the type and severity of skin lesions and represents an essential basis for antiviral and pharmacological research.

### 3.2. Viral Replication in Skin Cells

Once inside the cell, viruses take advantage of the host’s replication machinery to produce new viral particles. Viruses exploit host cellular systems for genome replication and virion production. They use membrane transport to migrate between compartments and to release virions [36,37].

The extracellular matrix (ECM) constitutes the first barrier to infection, and viruses can modify its integrity and function, including by activating inflammatory mediators. In addition to the ECM, viruses interact with the cell membrane and use endocytosis to enter the cell and ensure their replication [38,39]. Intracellular organelles, such as the endoplasmic reticulum, mitochondria, autophagosomes, and lysosomes, are remodeled to support the viral cycle [40].

Viruses induce the formation of biomolecular condensates as replication sites and can exploit the lysosomal system for exocytosis [41]. DNA viruses (HPV, HSV, VZV) largely use the nuclear machinery for transcription and replication, having replicative cycles well-adapted to keratinocyte differentiation [42]. RNA viruses (enteroviruses, parainfluenza viruses, measles) replicate in the cytoplasm and often produce a more intense inflammatory response. RNA viruses, including enteroviruses, measles, and parainfluenza viruses, replicate in the cytoplasm, often generating an intense inflammatory response through the activation of pattern-recognition receptors (PRRs) [43,44]. Some viruses with vascular tropism alter the dermal endothelium, contributing to the development of exanthemas and purpuric lesions. Disturbance of cutaneous homeostasis by interference with vascular proliferation, differentiation, or integrity leads to typical clinical manifestations: vesicles, papules and nodules, maculopapular exanthemas (measles), and vasculitic or necrotic eruptions in infections with endothelial tropism [45].

### 3.3. Local Immune Response of the Skin in Viral Infections

The skin is a complex immunological organ that functions both as a physical barrier and as an active platform for the recognition and elimination of pathogens. The local immune response plays an essential role in limiting the dissemination of viral infections and in maintaining tissue homeostasis [46].

The innate component of cutaneous immunity is triggered rapidly after contact with viral particles. Keratinocytes detect viral components through Toll-like receptors (TLRs) and RIG-I-like receptors, leading to the production of type I and III interferons, which inhibit viral replication and induce an antiviral status in neighboring cells [47].

In the dermis, Langerhans cells, dermal macrophages, and NK cells contribute to the initial control of infection through phagocytosis, interferon production, and elimination of infected cells. At the same time, inflammatory mediators such as IL-1, TNF-α, and various chemokines cause rapid recruitment of leukocytes to the site of infection, facilitating the formation of an active immune microenvironment [48].

Interferons stimulate the expression of Interferon-Stimulated Genes, a fundamental mechanism in the inhibition of DNA (e.g., HPV, HSV, VZV) and RNA viruses [46].

The adaptive immune response, particularly cytotoxic T lymphocytes, is essential for the elimination of persistent infections, while neutralizing antibodies limit viral spread [49].

Immunosuppressive conditions are associated with increased susceptibility to viral infections, enhanced viral replication, and more severe cutaneous manifestations [50]. Immunosuppressive therapy, prolonged corticosteroid use, and chronic inflammatory dermatitis further promote viral persistence and increase the severity of skin lesions.

### 3.4. Cytopathic Effects and Immune Modulation by Viruses

Viruses induce characteristic cytopathic changes in the skin, including ballooning degeneration, acantholysis, and vesicle formation in herpes infections; koilocytosis and epithelial hyperplasia in HPV infection; cytoplasmic viral inclusions in molluscum contagiosum; and vascular lesions in viruses with endothelial tropism [51].

To survive within host tissues, viruses employ multiple immune evasion strategies, such as inhibition of interferon production, downregulation of MHC I expression, induction of immune cells apoptosis, and establishment of latency [52]. These mechanisms predominantly affect MHC class I antigen presentation, limiting recognition by cytotoxic T lymphocytes, while indirectly interfering with MHC class II–dependent antigen presentation by antigen-presenting cells, resulting in an overall attenuation of both cellular and helper T-cell immune responses [53].

In herpesvirus infections, intranuclear replication underlies the typical cytopathic effects [54], whereas HPV modulates the keratinocyte cell cycle, as extensively documented [46,47]. Viruses with vascular tropism can affect the dermal endothelium, generating hemorrhagic or purpuric lesions [55].

These processes underline viral persistence, recurrent lesions, and atypical clinical manifestations observed in immunosuppressed patients or those with impaired cutaneous immunity.

## 4. Cutaneous Manifestations of DNA and RNA Viruses, Therapies, and Immunoprophylaxis

Viruses can be classified into two main types, RNA and DNA, based on genome composition. Each type replicates via specific enzymes, and the fidelity of these enzymes determines both the accuracy and efficiency of replication, maintaining the genetic integrity of the parental genome [56]. Table 1 provides a comparative synthesis of viruses involved in skin infections, their classification according to genome type, characteristic skin manifestations, and therapeutic approaches used. Cutaneous viral infections caused by DNA (HSV, VZV, HPV, MCV) and RNA (measles, rubella, parvovirus B19, Coxsackie) viruses cause a wide range of skin lesions, from papules and vesicles to maculopapular exanthemas, with complications that vary depending on the virus and the immune status of the host [49]. Rubella and Coxsackie viruses reach mucocutaneous surfaces by hematogenous dissemination after initial replication in the respiratory or gastrointestinal tract. While rubella causes predominantly immune-mediated lesions [57], Coxsackie viruses exhibit a direct epithelial tropism for the skin and mucous membranes, explaining the characteristic association between exanthems and enanthemas [58]. The enanthema in rubella infection and in Coxsackie virus infections differs in mechanism, clinical appearance, and diagnostic value. In rubella infection, the enanthema is usually discrete and transient, classically represented by Forchheimer spots (macules or punctate reddish spots on the soft palate and uvula), appearing early and the result of vascular damage and the mediated immune response [59]. In contrast, in Coxsackie virus infections, enanthema is frequent, clinically evident, and sometimes dominant, manifesting itself in painful blisters, erosions, or ulcerations on the oral mucosa (tongue, gums, jugular mucosa, palate), as a result of direct viral tropism for the epithelium of the mucosa and herpetic or local disease [60].

Antiviral therapy remains the standard for many skin infections: acyclovir, valacclovir, and famciclovir for HSV and VZV, specific immunoglobulins for exposures in vulnerable individuals, and adjuvant treatments for complications [61,62]. In chronic or persistent infections, such as HPV, therapeutic approaches include therapeutic vaccines and monitoring of premalignant lesions [63].

The development of new-generation vaccines, including mRNA platforms, plasmid DNA, and attenuated or recombinant viral vectors, allows for the induction of a rapid, robust, and specific immune response with increased safety [64]. mRNA vaccines have proven versatile and effective in stimulating humoral and cellular immunity at the skin level [65].

Modern adjuvants, immunomodulatory nanoparticles, liposomal emulsions, or polymeric systems enhance the immune response and direct protection to the skin, optimizing vaccination efficiency and reducing the required doses. The integration of genetic, epigenetic, and immunological data allows for the adaptation of vaccination schemes to individual susceptibility and the risk of skin complications [66]. Therapeutic vaccines represent a promising strategy against latent viruses (HSV, HPV), reducing recurrences and viral persistence [67]. Nanotechnologies and transcutaneous delivery systems (micro-needles, nanoparticle patches, bioactive hydrogels) allow for direct immunization at the skin level, increasing local immune response and patient compliance [68]. These platforms avoid traditional injectable administration and facilitate mass vaccination, representing an innovative direction with an impact on the prevention of viral skin infections.

**Table 1:** Cutaneous manifestations of viral infections and corresponding therapies.

Category	Virus	Virus Type	Cutaneous Manifestations	Treatment	References
Papulo-vesicular lesions	HSV Herpes Simplex	DNA ^1^	Grouped vesicles, ulcerations	Acyclovir, Valacyclovir, Isoprinosine’ Famciclovir	[69,70,71]
Papulo-vesicular lesions	VZV Varicella-Zoster Virus,	DNA	Typical vesicles, zoster	Acyclovir, Valacyclovir, Brivudin, Famciclovir	[72,73,74]
Papular lesions	MCV Molluscum Contagiosum Virus	DNA	Umbilicated papules	Curettage cryotherapy	[75,76]
Hyperplastic lesions	HPV Human Papilloma Virus,	DNA	Warts, dysplasia	Imiquimod, cryotherapy	[77,78]
Tumoral lesions	MCPyV Merkel cell polyomavirus	DNA	Merkel cell carcinoma	Surgery, immunotherapy	[79,80]
Vascular lesions	HHV-8 Human Herpesvirus	DNA	Kaposi sarcoma	ART (Antiretroviral Therapy)	[81,82]
Exanthems	Measles virus	RNA ^2^	Maculopapular exanthem	Supportive	[83]
Exanthems	Rubella virus	RNA	Fine exanthem	Supportive	[84,85]
Chronic infections	HCV Hepatitis C virus	RNA	Lichen planus, PCT (*Porphyria Cutanea Tarda*)	DAAs (Direct-Acting Antivirals)	[86]
Papulo-vesicular lesions	Parvovirus B19	DNA	Grouped vesicles, ulcerations	Acyclovir, Supportive	[87]

^1^ DNA—deoxyribonucleic acid, ^2^ RNA—ribonucleic acid.

### 4.1. DNA Viruses

DNA viruses are predominantly recognized by Toll-like 9. The main Toll-like receptor involved in the immune recognition of DNA viruses is Toll-like receptor 9, located in endosomal compartments, which detects unmethylated cytosine–guanine-rich double-stranded DNA sequences, characteristic of the viral genome and rare in eukaryotic DNA [88].

#### 4.1.1. Human Papilloma Virus (HPV)

HPV is a heterogeneous group of over 200 genotypes, of which approximately 40 infect the anogenital and oropharyngeal epithelia [89]. HPV is the most common sexually transmitted infection worldwide and constitutes a major public health challenge [90,91].

The presence of HPV in DNA in the saliva of patients with oral lichen planus supports the hypothesis of a possible cofactor role of viral infection in the pathogenesis of the disease. However, these results should be interpreted with caution and integrated into the context of the clinical, immunological, and therapeutic complexity that characterizes lichen planus, a condition with multifactorial etiopathogenesis [92,93].

HPV infection is transmitted by direct contact, often through skin microlesions. The mechanism of HPV infection is not fully elucidated. Recent studies have shown that the presence of cervical lesions associated with HPV infection increases the risk of contracting other sexually transmitted infections. In women, persistent HPV infection has been associated with an increased likelihood of HIV infection [67].

The most accepted model claims that the virus enters through microlesions of the epithelium, binds to the basement membrane, and is internalized by endocytosis. Subsequently, the viral genome is transported to the nucleus, where replication and transcription begin. The virus infects the basal cells of the epidermis, modifying the mechanisms of keratinocyte proliferation and differentiation. The result is epithelial hyperplasia and the formation of characteristic koilocytes [94].

High-risk genotypes (HPV 16, 18, 31, 33) induce malignant transformation through the oncoproteins E6 and E7, which inhibit the suppressor proteins p53 and Rb. At the cutaneous level, HPV is involved in squamous cell carcinoma, especially in immunocompromised patients or those chronically exposed to UV [46,95].

The use of prophylactic HPV vaccines began in 2006, targeting the immunization of adolescents. The first quadrivalent vaccine, which covers high-risk HPV types 16 and 18 and low-risk HPV types 6 and 11, was approved by the FDA in June 2006 for women aged 9–26 years [96]. The WHO launched a global strategy in 2020 to address cervical cancer as a public health problem. Recent population-based studies support single-dose HPV vaccination strategies, adopted in countries such as the United Kingdom and Spain, to increase coverage and reduce costs [97,98,99]. Evidence from Italy indicates a reduction in the HPV genotypes targeted by the vaccine [100]. However, disparities in access to healthcare continue to affect cervical cancer incidence among vulnerable women [101]. HPV is a non-enveloped DNA virus with cutaneous and mucosal tropism, implicated in benign warts, condylomas, precancerous lesions, and, in persistent cases, malignant transformation. The viral proteins E6 and E7 interfere with the cell cycle by inhibiting p53 and Rb, promoting keratinocyte proliferation and genomic instability, especially in persistent infections with oncogenic risk genotypes [102].

Prophylactic vaccines (Cervarix, Gardasil, Gardasil 9) use virus-like particles (VLPs), induce neutralizing antibodies against the L1 capsid protein, and provide protection against oncogenic strains and those causing genital warts [94,103]. Vaccines do not eliminate existing infections, but they can reduce the recurrence of lesions and the severity of symptoms [104].

To date, there are no standardized anti-HPV immunoglobulins; active therapy remains based on local and ablative methods. HPV vaccination has the potential to prevent skin and mucosal lesions, but data on cutaneous beta and gamma strains are limited [105]. Major obstacles include vaccination coverage, population reluctance, and lack of inclusion in national schemes [106]. Future directions include the development of therapeutic vaccines (based on E6/E7 proteins), the evaluation of protection against cutaneous genotypes, increasing population coverage, and implementing public health strategies to reduce the incidence of HPV-associated lesions and cancers [94,107].

Herpesviruses play an important role in certain dermatoses: HHV-8 is involved in the pathogenesis of Kaposi’s sarcoma by stimulating angiogenesis and cell proliferation, and Pityriasis rosea Gibert is associated with HHV-6/HHV-7 reactivation in an immune and epigenetic context, supported by both clinical and molecular data [108].

HPV is a viral agent with high pathogenetic plasticity. Vaccination prophylaxis is the most robust protection, while immunological and therapeutic therapies remain under investigation [109]. Expanding research and vaccination is essential to prevent persistent skin and mucosal infections and oncogenic complications [110]. Innovative approaches, such as microbiome modulation, therapeutic vaccines, and liquid biopsy biomarkers, are emerging as promising prospects. HPV infection has major clinical implications due to its association with cutaneous and mucosal lesions, precancerous changes, and HPV-related malignancies [111]. Prophylactic vaccination remains the most effective preventive strategy, while limited therapeutic options highlight the importance of screening, increased vaccine coverage, and the development of targeted therapeutic vaccines [112].

#### 4.1.2. Herpes Simplex Virus (HSV-1/HSV-2)

HSV-1 and HSV-2 are neurotropic viruses with the ability to establish latency in sensory ganglia. Cold sores occur through reactivation of HSV-1, causing blisters grouped on an erythematous base, followed by crusts [113,114]. Genital herpes is more commonly associated with HSV-2, manifested by painful blisters, erosions, and systemic symptoms [115]. Pathogenesis by replication in keratinocytes causes ballooning degeneration, acantholysis and blister formation. Reactivation is favored by stress, fever, UV exposure and immunosuppression. Complications are erythema multiforme, eczema herpeticum, herpetic keratitis [116,117].

HSV 1 and HSV 2 are DNA viruses of the *Herpesviridae* family, responsible for skin and mucosal infections (cold sores and genital herpes) worldwide [118]. The virus infects the squamous epithelium after contact with infected secretions or microcracks in the skin/mucosal membrane and replicates locally, generating blisters or ulcers. HSV subsequently migrates to the sensory nerve ganglia, where it persists latently, and can be reactivated by factors such as stress, trauma, or immunosuppression [118,119]. The molecular mechanisms underlying HSV entry into keratinocytes involve the initial attachment of the virus to 3-O-sulfated heparan sulfate proteoglycans, followed by the engagement of key entry receptors, Nectin-1 and the herpesvirus entry mediator, which mediate viral membrane fusion and subsequent viral internalization [120].

There is currently no licensed vaccine against HSV, although several experimental candidates are under study [121]. Standard antiviral therapy involves nucleoside analogs (acyclovir, valacyclovir), which reduce the duration of lesions and contagiousness, but do not eliminate latent virus [95]. Passive therapy with specific anti-HSV immunoglobulins is not used in clinical prevention or treatment.

Prevention of skin and mucosal infections is based on behavioral measures: avoiding contact during active lesions, using condoms, and maintaining hygiene; suppressive antiviral therapy can reduce recurrences and transmissibility [95,122]. Future research aims to develop vaccines and therapies that reduce recurrences and prevent primary infections, with a major impact on public health [119]. HSV infections have major clinical relevance due to their high prevalence, lifelong latency, and frequent mucocutaneous recurrences [123]. Although current antiviral therapies reduce disease severity and transmission, they do not eradicate latent infection, highlighting the need for effective preventive strategies and HSV vaccine development [124].

#### 4.1.3. Varicella-Zoster Virus (VZV)

VZV is a neurotropic DNA herpesvirus responsible for primary infection (chickenpox) and latent reactivation as shingles. The virus enters the respiratory tract or by direct contact with infected secretions, initially replicating in the epithelium and causing viremia and cutaneous dissemination. The characteristic rash typically progresses through macules, papules, vesicles, and crusts [125,126].

Varicella is characterized by a polymorphic, pruritic rash with lesions in varying stages of development. The disease is mostly self-limited in children. Complications include bacterial superinfections, thrombocytopenia, and neurological damage in immunocompromised patients [127]. VZV-specific T-cell-mediated immunity is essential for maintaining viral latency and preventing the development of herpes zoster [128].

After initial infection, VZV remains latent in peripheral ganglion neurons, and virus-specific memory T cells periodically control subclinical reactivation. Decline in T-cell immunity with age favors reactivation and the development of clinical disease [129]. Herpes zoster does not result from external infection, but can be transmitted by contact with people infected with varicella or shingles [130].

The virus remains latent in sensory ganglia, and reactivation may be facilitated by advanced age, stress, or immunodeficiency. This results in dermatomal eruptions with neuropathic pain. Complications include postherpetic neuralgia and bacterial superinfections [131,132].

Vaccination is the primary method of prevention. For varicella, a live attenuated virus vaccine (Varivax/Varilrix) is used, administered at 12–15 months, with a booster at 4–6 years [133,134].

For the prevention of reactivation in adults and the elderly, the vaccines Zostavax (live attenuated virus) and Shingrix (recombinant subunit adjuvanted) are available, the latter offering longer-lasting protection and superior efficacy. Post-exposure administration can reduce the risk of disease by approximately 80% if the vaccine is administered within the first 3 days after contact [135,136].

Vulnerable immunocompromised individuals, newborns, premature infants, or pregnant women without immunity can benefit from specific anti-VZV immunoglobulins (VariZIG) for post-exposure prophylaxis, reducing the risk of severe forms [137,138].

Active treatment of the infection includes antivirals (acyclovir, valacyclovir), which reduce the severity and duration of lesions, but do not eliminate the latent virus [139]. VZV infection has major clinical implications due to its ability to establish neuronal latency and reactivate as herpes zoster, particularly in elderly and immunocompromised individuals [140]. Vaccination is the most effective preventive strategy, while antiviral therapy reduces disease severity and complications without eliminating viral latency [141].

#### 4.1.4. Molluscum Contagiosum Virus (MCV)

Molluscum contagiosum is a viral skin infection caused by MCV, a double-stranded DNA poxvirus, the largest virus that infects humans. Of the four known genotypes (MCV-1–MCV-4), MCV-1 causes the majority of infections (76–97%) [142]. MCV causes benign lesions, common in children, adults with atopic dermatitis, or immunocompromised individuals. Typical lesions are cup-shaped, shiny papules with a central umbilication [143]. Viral replication in the keratinocytes of the granular layer forms the characteristic Henderson–Patterson inclusions. Transmission occurs as a result of direct contact, fomites; in adults, it is often transmitted by sexual contact. With regard to features of the virus, lesions may be numerous and persistent in HIV-positive patients [144].

MCV is a DNA virus of the *Poxviridae family* that infects epidermal keratinocytes, causing papulonodular, umbilicated lesions, contagious through direct contact or contaminated objects. The virus produces proteins that inhibit the local immune response, favoring persistence and autoinoculation; the infection typically lasts 6–18 months in immunocompetent individuals [24,145].

There is currently no licensed vaccine for MCV. There are no specific antibodies or immunoglobulins for MCV [146,147]. Treatment includes physical/topical methods: curettage, cryotherapy, and topical substances, but also modern therapies such as Berdazimer sodium (Zelsuvmi) or antivirals (Cidofovir) for immunocompromised patients. Intralesional immunotherapy with non-specific antigens has demonstrated clearance of lesions [148,149].

In the absence of a vaccine, prevention is based on hygiene and behavioral measures: covering lesions and avoiding scratching and sharing objects. In high-risk patients, topical therapies, antivirals, or immunotherapy can reduce persistence and transmission [150,151]. Because this virus can be sexually transmitted, the patient should be encouraged to practice safe sex and use barrier methods for protection. MCV infection has clinical relevance due to its high contagiousness and the persistence of lesions, particularly in children and immunocompromised patients [152]. In the absence of a vaccine or virus-specific therapy, clinical management relies on local treatments, immunomodulatory approaches, and hygienic–behavioral preventive measures to reduce transmission [153].

#### 4.1.5. Polyomaviruses (HPyV): Merkel Cell Carcinoma

Polyomaviruses (HPyV) are a family of non-enveloped viruses with a double-stranded circular DNA genome of approximately 5000 base pairs [154]. First described in 2008, MCPyV (or human polyomavirus 5) is a small DNA virus recognized as the main causative agent of Merkel cell carcinoma (MCC), a rare but aggressive neuroendocrine skin cancer [155], characterized by rapid proliferation, early metastasis, and high mortality. Etiologically, in most (virus-positive) cases, the disease is associated with Merkel cell polyomavirus (MCPyV) [156,157].

At the molecular level, MCPyV clonally integrates into the tumor cell genome. In positive tumors, the viral “early” region expresses the oncoprotein antigens, small T antigen (sTAg) and truncated large T antigen (LTAg-t); truncation of LTAg is a constant element, maintaining only the pRb binding domain and losing the helicase domain necessary for autonomous viral replication [158,159].

Functionally, sTAg appears to be the driver of malignant transformation, with a 2024 study demonstrating that sTAg is more efficient than LTAg-t at cellular transformation and localizes to the nucleus even without a classical nuclear signal. The mechanism of transformation involves recruitment of the MYCL–EP400 complex by sTAg, with downstream dysregulation including TP53 inactivation and cell cycle activation [159,160,161].

Thus, MCPyV-mediated oncogenesis is considered a two-step process: (1) clonal genomic integration of MCPyV and (2) mutations/truncations in LTAg that prevent viral replication but promote malignant transformation [162,163].

From a clinical–epidemiological point of view, MCPyV infection is very common; most individuals are benign, asymptomatic carriers [164]. Only a small fraction develops MCC, under the influence of predisposing factors: advanced age, immunosuppression, and/or chronic exposure to UV radiation [165].

Recently, detection of MCPyV using a monoclonal antibody to LTAg and optimized qPCR showed the virus in 97% of tumors, according to Rodig et al., suggesting that almost all MCCs could be virus-associated. This has implications for classification, prognosis, and therapies [162].

It has been shown that patients with MCC develop T and B responses specific to viral antigens. This justifies the use of immunotherapy (blockade of the PD-1/PD-L1 checkpoint) and provides the basis for the development of future therapies: therapeutic vaccines and adoptive T-cell therapies (TCR-T) [163].

A recent observational study showed that the presence of serum anti-MCPyV oncoprotein antibodies (“AMERK” test) at diagnosis is associated with better survival and relapse in patients with localized disease [165].

However, not all MCCs are MCPyV-positive. MCPyV-negative tumors occur more frequently in immunosuppressed patients and are attributed to UV-induced mutations, which are common in tumor surveillance genes (TP53, RB1). The tumor microenvironment, including mast cell density, may influence tumor aggressiveness, independent of viral status. Also, PD-L1 expression does not always correlate with MCPyV status or survival. In the therapeutic term, sTAg remains a central “driver” of transformation and a primary target for future immunological or antiviral therapies. MCC associated with MCPyV represents a paradigm of viral oncogenesis with major clinical implications for diagnostic accuracy, prognostic stratification, and therapeutic decision-making [166]. Assessment of MCPyV status supports the use of immunotherapy and identifies the sTAg oncoprotein as a key molecular target for future personalized treatment strategies [167].

### 4.2. RNA Viruses

RNA viruses are detected by a complex network of TLRs specialized in RNA recognition. The main mechanism of immune recognition of RNA viruses involves endosomally located Toll-like receptors, in particular Toll-like receptors 3, 7, and 8, which detect the presence of single-stranded or double-stranded viral RNA resulting during replication, triggering the activation of signaling pathways responsible for the induction of type I interferons and the antiviral inflammatory response [168].

#### 4.2.1. Measles Virus: Maculopapular Exanthemas

The measles virus, placed in the genus *Morbillivirus*, family *Paramyxoviridae*, and subfamily *Orthoparamyxovirinae*, is highly contagious and produces marked systemic and cutaneous manifestations. The virus is characterized by exanthema with confluent maculopapular rash and cephalo-caudal distribution [169,170]. Pathognomonic elements are Koplik spots in the oral cavity. Viral complications include superinfections, encephalitis, and subacute sclerosing panencephalitis [171]. Approximately 90% of people who come into contact with the virus and who do not have immunity will develop symptoms [172].

Measles virus is an RNA paramyxovirus, highly contagious, that infects respiratory epithelial cells and lymphocytes, causing viremia and systemic dissemination. The typical cutaneous manifestation is a maculopapular rash, which develops in waves, initially on the face and trunk, associated with fever, cough, rhinorrhea, and conjunctivitis [49].

The live attenuated MMR vaccine (measles, mumps, and rubella) is highly effective for preventing measles. The recommended schedule includes two doses, the first at 12–15 months and a booster at 4–6 years. Post-exposure vaccination within the first 72 h can prevent or attenuate the disease. In case of exposure to the virus or in immunocompromised patients, the administration of specific immunoglobulins (IGIV) can reduce the severity or prevent the disease [173,174].

Routine vaccination remains the main method of preventing measles and its complications. Immunoglobulins are indicated for post-exposure prophylaxis in vulnerable individuals. Maintaining high vaccination coverage in the population prevents epidemics and protects groups at risk [175]. Measles virus infection has major clinical implications due to its extremely high contagiousness and the risk of severe systemic and neurological complications [176]. The MMR vaccination remains the most effective preventive strategy, while post-exposure vaccination or immunoglobulin administration is crucial for protecting vulnerable individuals and preventing outbreaks [177].

#### 4.2.2. Rubella Virus: Rash and Lymphadenopathy

Rubella is an acute, contagious infection caused by an RNA virus of the *genus Rubivirus* of the family *Togaviridae*, which is spread by airborne droplets when infected people sneeze or cough [178].

Rubella infection during pregnancy can be serious, leading to spontaneous abortion, intrauterine fetal death, or severe congenital malformations (cataracts, congenital heart disease, hearing impairment, and developmental delays) including congenital rubella syndrome (CRS). Manifestations include discrete maculopapular rash, occipital and retroauricular lymphadenopathy, and arthralgias [179].

Rubella virus is an RNA togavirus that infects epithelial and endothelial cells, eliciting a systemic immune response and characteristic skin manifestations (maculopapular rash) and lymphadenopathy [180]. The rash begins on the face, spreads to the trunk and extremities, and is associated with mild fever and minimal respiratory symptoms [172,181].

The live attenuated MMR vaccine prevents rubella infection, with a two-dose schedule recommended at 12–15 months and a booster at 4–6 years. Vaccination of women of childbearing age is crucial for the prevention of congenital rubella syndrome. Antibody and immunoglobulin therapy is used; there is no specific antiviral treatment. Immunoglobulins can be used for post-exposure prophylaxis in unimmunized pregnant women to reduce the risk of fetal infection [180,182].

In terms of prevention, maintaining high vaccination coverage prevents cases and protects vulnerable groups. Infection control relies on universal immunization, identification and isolation of suspected cases, and serological monitoring of pregnant women. Rubella virus infection has major clinical implications due to its severe consequences during pregnancy and its role as a preventable cause of congenital rubella syndrome [183]. The MMR vaccination remains the cornerstone of prevention, while serological monitoring and post-exposure prophylaxis are essential for protecting women of childbearing age and fetal health [184].

#### 4.2.3. Parvovirus B19

Parvovirus B19 is a small DNA virus that infects erythroblasts in the bone marrow and endothelial cells, causing viremia and a systemic immune response [185]. Cutaneous manifestations include a slapped-cheek facial rash and a maculopapular rash on the trunk and extremities. In adults, arthralgia and transient arthritis may occur [186].

Parvovirus B19 predominantly affects children. The typical presentation is a slapped-cheek facial rash followed by a reticulated rash on the trunk and limbs [187]. Mechanisms by which the virus infects erythroid precursors cause aplasia in patients with hemoglobinopathies. There is currently no licensed vaccine for Parvovirus B19. Recent research is investigating candidate vaccines based on the VP1/VP2 capsid protein [188].

There is no specific antiviral treatment. Intravenous immunoglobulin (IVIG) can be used in immunocompromised patients to prevent or alleviate severe symptoms [31,189].

The main strategy is to avoid contact with infected individuals, especially in schools and childcare centers. In vulnerable patients (pregnant, immunocompromised), immunoglobulin prophylaxis may reduce the risk of complications [190]. Parvovirus B19 infection has clinical relevance due to its characteristic cutaneous manifestations, the risk of severe anemia in patients with underlying hematologic disorders, and its potential impact during pregnancy [191]. In the absence of a vaccine or specific antiviral therapy, clinical management relies on early recognition, monitoring of vulnerable populations, and the use of immunoglobulins in severe cases [192].

#### 4.2.4. Coxsackie Virus: Hand-Foot-Mouth Disease

Coxsackievirus A6 (CVA6) has become increasingly clinically relevant as a cause of hand-foot-and-mouth disease (HFMD) worldwide since 2008. Enteroviruses, particularly Coxsackie A16 and EV71, cause a contagious, eruptive infection [193]. Lesions present as blisters on the palms, soles, and oral mucosa, and moderate fever. Complications are rare, but EV71-associated forms can cause neurological damage. The disease is self-limited but communicable in communities [194].

Coxsackieviruses A and B, members of the *Picornaviridae family*, are RNA viruses that infect epithelial cells and lymphocytes, causing viremia and cutaneous manifestations. Clinically, the disease is characterized by vesicular and ulcerative lesions on the hands, feet, and oral cavity, sometimes associated with fever and malaise [195]. In Coxsackie virus infections, enanthema is frequent, clinically evident, and sometimes dominant, manifesting as painful blisters, erosions, or ulcerations on the oral mucosa (tongue, gums, buccal mucosa, palate) [196].

Currently, inactivated vaccines against Coxsackievirus A16 and enterovirus 71 (EV71) have been developed and used in China, which reduce the incidence of severe HFMD [197,198]. Globally, the vaccine is not yet universally available.

There is no specific antiviral treatment for HFMD. Intravenous immunoglobulins (IVIG) can be used in severe cases in immunocompromised patients or those with neurological complications [199,200].

Rigorous hygiene measures, isolation of infected children, and avoidance of contact with infected individuals are essential to control the spread of the virus. Available vaccines can prevent severe forms and complications [194]. Coxsackievirus infection is clinically important due to its high transmissibility and its impact on pediatric communities, particularly in HMFD [201]. Although the disease is usually self-limited, EV71-associated forms may lead to severe neurological complications, emphasizing the need for preventive measures, epidemiological control, and broader access to effective vaccines.

#### 4.2.5. Hepatitis C Virus (HCV): Associated Dermatological Manifestations

HCV infection represents a major public health issue of paramount global importance. HCV is a spherical, enveloped virus belonging to the *Flaviviridae family*. HCV is a hepatotropic RNA virus, but it also produces significant extrahepatic effects, including immune-mediated cutaneous manifestations [202,203]. Chronic HCV infection leads to a wide range of dermatological and vasculitic conditions such as lichen planus, hypertrophic lichen planus, mixed cryoglobulinemia, porphyria cutanea tarda, cutaneous vasculitis, and chronic pruritus [204,205].

HCV-associated skin lesions are immune-mediated, through T-lymphocyte activation, production of pro-inflammatory cytokines, and deposition of circulating immune complexes in the dermis and vascular endothelium [206]. These mechanisms explain both the lichenoid aspect of the lesions and the vasculitic phenomena and chronic pruritus. In mixed cryoglobulinemia, HCV–antibody immune complexes deposit in the vessels, producing inflammation and endothelial necrosis, which lead to purpura and cutaneous ulceration [207].

There is currently no licensed vaccine against HCV. Ongoing research focuses on developing preventive vaccines based on structural and non-structural viral proteins, with the aim of preventing chronic infection and extrahepatic complications [208]. Likewise, no immunoglobulins or specific antibodies for HCV are available.

Standard treatment relies on direct-acting antivirals (DAAs), which reduce viral replication and can lead to the improvement of secondary cutaneous manifestations [202]. Recent studies show that eradication of HCV with DAAs may result in resolution or improvement of lichen planus, vasculitis, and porphyria cutanea tarda [209].

Preventive strategies are based on controlling HCV transmission through screening and reducing parenteral exposure, as well as on early antiviral treatment to prevent the onset or persistence of dermatological lesions. Thus, the integrated management of the patient with HCV should include dermatological evaluation and prompt antiviral therapy to prevent and improve cutaneous complications. HCV infection has major clinical implications due to its immune-mediated extrahepatic cutaneous manifestations, which may precede or accompany chronic liver disease [210]. Direct-acting antiviral therapy not only eradicates the virus but can lead to the improvement or resolution of dermatological lesions, highlighting the importance of early diagnosis and integrated interdisciplinary management [211].

#### 4.2.6. SARS-CoV-2 Virus

The SARS-CoV-2 virus, responsible for COVID-19, is an RNA virus with a lipid envelope and belongs to the *Coronaviridae family*, genus *Betacoronavirus*. The disease caused by SARS-CoV-2 infection is primarily a respiratory condition, but numerous skin manifestations have been reported that may occur concurrently with respiratory symptoms, before them, or even in isolation [212,213]. The most common skin patterns associated with COVID-19 are chilblain-like lesions (CBLLs), maculopapular lesions, urticarial lesions, vesicular lesions, and livedoid lesions. These include several clinical patterns: chilblain-like lesions (“COVID toes”), common in young patients and children, usually associated with mild forms of the disease [214]; maculopapular rashes, characterized by erythematous spots and papules on the trunk and limbs; and urticarial lesions, with pruritic erythematous plaques, which may precede or accompany respiratory symptoms [215]. There may also be vesicular eruptions, similar to those seen in chickenpox. Livedo reticularis or vascular lesions (purpura, petechiae) suggest microvascular involvement. In a study in Romania, Tatu et al. report a case of a family cluster of a maculopapular COVID-19 rash. Eight people had COVID-19 symptoms, six were confirmed by SAR-CoV-2 immunoluminescent tests, and the four associated with blood tests had skin manifestations [212]. Rarer manifestations have also been reported, such as erythema multiforme lesions, signs associated with multisystem inflammatory syndrome in children (MIS-C) or adults (MIS-A) [214]. Recognizing these manifestations is important because skin rashes can sometimes be the first sign of infection, facilitating early diagnosis. Human angiotensin-converting enzyme 2 expression on keratinocyte is demonstrated to be a possible entry point for SARS-CoV-2 [216,217].

Skin manifestations associated with SARS-CoV-2 infection are, in most cases, self-limiting and do not require specific systemic treatment, and management is generally symptomatic and aimed at alleviating discomfort. In mild forms of maculopapular, urticarial, or vesicular rashes, medium- to high-potency topical corticosteroids are recommended to reduce local inflammation, and oral antihistamines are recommended to control pruritus [218]. In some cases, with extensive lesions or severe pruritus, the use of systemic corticosteroids may be considered, with the precaution of avoiding their administration in the acute phase of the viral disease, as they may prolong viral clearance. For persistent or severe manifestations, such as extensive vasculopathies or livedo-type lesions, immunomodulators such as cyclosporine or intravenous immunoglobulins have been used in some reports, but evidence remains limited and is based more on clinical experience than on randomized controlled trials.

It is also important to rule out other causes of skin lesions, such as drug reactions or manifestations of pre-existing dermatological diseases, as these may influence the choice of treatment [219]. Correct diagnosis of the type of lesion—maculopapular, urticarial, vesicular, or chilblain-like—can also guide the therapeutic decision, as many skin lesions associated with COVID-19 are transient and resolve without specific therapy.

In terms of prevention, the main strategies remain linked to preventing SARS-CoV-2 infection. COVID-19 vaccination significantly reduces the risk of infection and, implicitly, the incidence of skin manifestations associated with active disease [220]. General preventive measures include physical distancing, wearing masks in crowded spaces, and rigorous hand hygiene, which reduce viral transmission and, implicitly, the risk of skin manifestations associated with infection. SARS-CoV-2 infection has relevant clinical implications due to the wide spectrum of associated cutaneous manifestations, which may occasionally represent the first or only sign of disease [221]. Early recognition of these lesions supports timely diagnosis and appropriate patient management, while vaccination remains the primary preventive strategy, reducing the risk of severe disease and COVID-19-related skin manifestations [222].

## 5. Future Directions and Current Limitations in Dermatovirology Research

Dermatovirology is a dynamic field, located at the interface of dermatology, immunology, and molecular biology, with a key role in understanding cutaneous viral infections. Future research should focus on clarifying the interactions between viruses and the skin barrier, in particular on the role of the microbiome and epidermal dysfunction in the susceptibility to viral infections. Identification of host factors involved in local immune control and the development of advanced experimental models, such as cutaneous organoids and reconstructed human skin, are essential for studying pathogenetic mechanisms and for testing antiviral therapies and vaccines. The development of topical antivirals and innovative delivery systems, such as nanoparticles or smart hydrogels, represents a promising direction for increasing therapeutic efficacy and reducing adverse effects, and it is necessary to expand prophylactic and therapeutic vaccines for viruses.

Despite recent advances in dermatovirology, the present study has several limitations. The complexity of interactions between viruses, the cutaneous barrier, and local immune responses limits the direct translation of experimental findings into clinical practice. Current experimental models, including cell cultures, reconstructed human skin, and cutaneous organoids, do not fully capture the heterogeneity of human skin or the impact of systemic factors. Moreover, the role of the cutaneous microbiome in viral susceptibility and immune modulation remains incompletely understood, being strongly influenced by genetic and environmental variability. Most topical antiviral strategies and innovative delivery systems are still at a preclinical stage, and the lack of large-scale clinical trials restricts long-term evaluation of their efficacy and safety. Finally, the limited availability of prophylactic and therapeutic vaccines for skin-tropic viruses continues to constrain effective prevention and disease control.

## 6. Conclusions

Cutaneous viral infections represent a heterogeneous group of disorders arising from complex interactions between viral pathogens, the skin barrier, and the host immune response. This review highlights that the clinical spectrum of viral skin manifestations is strongly influenced by viral genome type (DNA versus RNA), cellular tropism, and mechanisms of immune evasion or persistence within cutaneous tissues. DNA viruses, including HPV, HSV, VZV, MCV, and polyomaviruses, are frequently associated with persistent or latent infections, leading to recurrent, hyperproliferative, or neoplastic skin lesions, particularly in immunocompromised individuals. In contrast, RNA viruses more commonly induce acute, self-limited exanthematous eruptions that reflect systemic viral dissemination and robust innate immune activation. Understanding these pathogenetic differences is essential for accurate diagnosis, risk stratification, and tailored clinical management. Advances in antiviral therapy and immunoprophylaxis have significantly improved outcomes in many cutaneous viral infections. Vaccination remains the most effective preventive strategy, while novel therapeutic approaches, including therapeutic vaccines, mRNA-based platforms, immune modulators, and targeted antiviral agents, offer promising avenues for controlling chronic or recurrent infections. Emerging transcutaneous and nanotechnology-based delivery systems further enhance the potential for skin-directed immunization and improved patient compliance. Overall, an integrated understanding of viral tropism, cutaneous immune responses, and host susceptibility is critical for optimizing prevention and treatment strategies. Future research should focus on personalized immunoprophylactic approaches, identification of predictive biomarkers, and development of innovative antiviral therapies to reduce the global burden of viral skin diseases.

## Data Availability

The data presented in this study are available on request from the corresponding author.

## Abbreviations

The following abbreviations are used in this manuscript:

HSV-1	Herpes Simplex Virus Type 1
HPV	Human Papilloma Virus
MCV	Molluscum Contagiosum Virus
HSV	Herpes Simplex Virus
VZV	Varicella-Zoster Virus
ECM	The extracellular matrix
PRRs	pattern-recognition receptors
TLR	through Toll-like receptors
RNA	ribonucleic acid
DNA	deoxyribonucleic acid
ART	Antiretroviral Therapy
DAAs	Direct-Acting Antivirals
HPyV	Polyomaviruses
MCC	Merkel cell carcinoma
MCPyV	Merkel cell polyomavirus
LTAg-t	truncated large T antigen
sTAg	small T antigen
TCR-T	adoptive T-cell therapies
MMR	vaccine (measles, mumps, and rubella)
IGIV	specific immunoglobulins
CRS	congenital rubella syndrome
IVIG	Intravenous immunoglobulin
CVA6	Coxsackievirus A6
HFMD	hand-foot-and-mouth disease
EV71	Enteroviruses 71
HCV	Hepatitis C virus
WHO	World Health Organization
ECDC	Centre for Disease Prevention and Control
